# Disparities in Emergency Medical Services Intra-Arrest Transport by Neighborhood Socioeconomic Vulnerability

**DOI:** 10.1001/jamanetworkopen.2026.3764

**Published:** 2026-04-03

**Authors:** Meghan M. Hewlett, Remle P. Crowe, Emily E. Ager, James S. Ford, Mary P. Mercer, Renee Y. Hsia

**Affiliations:** 1 Department of Emergency Medicine, University of California, San Francisco, San Francisco; 2 ESO Inc, Austin, Texas; 3 Department of Emergency Health Sciences, University of Texas Health San Antonio, San Antonio; 4 Department of Emergency Medicine, University of California, San Diego, San Diego

## Abstract

**Importance:**

Out-of-hospital cardiac arrest (OHCA) survival is lower in neighborhoods with low (vs high) socioeconomic status. While emergency medical services (EMS) practices of intra-arrest transport (IAT) vary, it is unknown whether neighborhood-level factors are associated with these transport patterns.

**Objective:**

To determine the association between greater neighborhood socioeconomic vulnerability and odds of IAT among adults with OHCA.

**Design, Setting, and Participants:**

This cohort study used a large national database of deidentified EMS electronic health record data. The cohort comprised US adults (aged ≥18 years) with attempted EMS resuscitations for nontraumatic OHCA between January 1, 2022, and December 31, 2022. Data were analyzed between December 2023 and December 2024.

**Exposures:**

The exposure was the Social Vulnerability Index (SVI) of the EMS encounter within a US Census tract. Neighborhoods were stratified into quartiles based on SVI percentile. Higher SVI percentiles indicated greater socioeconomic vulnerability.

**Main Outcome and Measure:**

The primary outcome was the odds of IAT. The association between SVI quartile and EMS transport pattern was assessed using marginal logistic regression modeling.

**Results:**

A total of 61 524 patient encounters were included. Patients had a median (IQR) age of 65 (52-76) years and included 38 546 males (62.6%). Bystander resuscitation was administered in 23 124 encounters (37.6%), while 23 492 patients (38.2%) received IAT and 38 032 patients (61.8%) received continued on-scene resuscitation. Adults in neighborhoods in the highest SVI quartile had a higher occurrence of IAT than those in the lowest SVI quartile (7052 [41.1%] vs 4000 [32.8%]; *P* < .001). After adjustment, patients in the highest SVI quartile had greater odds of receiving IAT than those in the lowest SVI quartile (adjusted odds ratio, 1.35; 95% CI, 1.15-1.57).

**Conclusions and Relevance:**

In this cohort study of EMS resuscitations of adults for nontraumatic OHCA, greater neighborhood socioeconomic vulnerability was associated with increased odds of IAT. This association may contribute to socioeconomic disparities in OHCA outcomes and warrant further investigations into factors in EMS transport decisions for OHCA, particularly in marginalized communities.

## Key Points

**Question:**

Is neighborhood socioeconomic vulnerability index (SVI) associated with emergency medical services (EMS) intra-arrest transport for adults with out-of-hospital cardiac arrest (OHCA)?

**Findings:**

In this cohort study of 61 524 patient encounters for nontraumatic OHCA, patients in neighborhoods with the most vulnerable SVI quartile had greater adjusted odds of receiving intra-arrest transport than patients in neighborhoods with the least vulnerable SVI quartile.

**Meaning:**

The findings suggest that EMS transport practices for OHCA may contribute to socioeconomic disparities in OHCA outcomes.

## Teaser

This cohort study explores whether neighborhood Social Vulnerability Index is associated with emergency medical services (EMS) practice of intra-arrest transport among adults with nontraumatic out-of-hospital cardiac arrest.

## Introduction

Socioeconomic disparities in out-of-hospital cardiac arrest (OHCA) outcomes are well documented, with neighborhoods of lower socioeconomic status having lower survival and lower likelihood of neurologically favorable outcomes.^1,2,3,4,5,6,7^ While contributing factors have been proposed, such as lower rates of bystander cardiopulmonary resuscitation (CPR), lower use of automated external defibrillator devices, and longer emergency medical services (EMS) response times, these factors do not fully account for observed disparities.^6,7,8,9,10^ There is a need to identify other points along the OHCA chain of survival that potentially contribute to socioeconomic disparities in OHCA outcomes. Emerging evidence suggests that EMS decision-making during resuscitation plays an important role.^11,12,13,14,15^

Intra-arrest transport (IAT), defined as the practice of transporting patients during ongoing resuscitation for OHCA, has been associated with lower survival compared with continued on-scene resuscitation (COSR).^16,17^ IAT may lead to interruptions in CPR and impair CPR quality, particularly during ambulance departure, thus potentially worsening patient survival and neurologic outcomes.^18,19,20,21^ Moreover, in studies examining outcomes in extracorporeal CPR, the benefit of IAT over COSR is not definitive.^22,23^ Despite these concerns, IAT remains common, and no widely accepted, comprehensive EMS guidelines address IAT practices.^24^ While IAT practices vary widely across EMS systems, limited studies have explored whether IAT practices differ by patient demographic characteristics.

**Figure 1.**
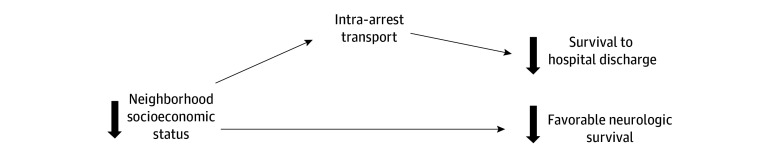
Modeling of Interplay Between Intra-Arrest Transport, Neighborhood Socioeconomic Status, and Out-of-Hospital Cardiac Arrest Outcomes

Nonclinical factors may affect EMS resuscitation and transport decisions. However, the relationship between neighborhood socioeconomic status and IAT has not been examined (Figure 1). Therefore, this study aimed to determine the association between neighborhood socioeconomic vulnerability and odds of IAT among adults with OHCA.

## Methods

### Study Design and Setting

We conducted a retrospective cohort study using deidentified EMS electronic health record (EHR) data for US adults with OHCA. Data were obtained from the 2022 ESO Data Collaborative public release research dataset (ESO Inc), which includes records from more than 3000 EMS agencies. These agencies voluntarily agreed to have their deidentified records included in the dataset. The University of California, San Francisco Institutional Review Board deemed this study exempt from review and waived the informed consent requirement because deidentified data were used. We followed the Strengthening the Reporting of Observational Studies in Epidemiology (STROBE) reporting guideline.^25^

EMS records are stripped of patient, EMS clinician, and EMS agency identifiers before inclusion in the dataset. Records include EMS-collected patient demographic characteristics and time-stamped events and interventions; this collection is in adherence to the National EMS Information System (NEMSIS) standard.^26^ These data and collection procedures are detailed elsewhere.^27,28,29,30,31^

Scene location data were reported at the US Census tract level, a commonly used proxy for neighborhoods, as a tract contains up to 8000 residents and is socioeconomically homogenous.^29^ Socioeconomic vulnerability was assessed using the Social Vulnerability Index (SVI) of the Centers for Disease Control and Prevention and the Agency for Toxic Substances and Disease Registry, which is linked to scene location by ESO as an honest broker. Geographic identifiers were subsequently removed to maintain deidentification. We selected the year 2022 to minimize confounding from COVID-19 pandemic–related disruptions to EMS utilization and case mix in the US. During 2022, EMS utilization decreased and the demographic composition of calls shifted toward older patients, rural areas, and higher call severity (eg, higher transport rates and field deaths).^32,33^

### Inclusion of Cardiac Arrest Encounters

Our cohort included encounters for EMS-treated OHCA, occurring prior to EMS arrival, in adults (aged ≥18 years) between January 1, 2022, and December 31, 2022. We excluded encounters for OHCA that involved the following: EMS responses to mass casualty incidents; air or water rescues; incidents occurring at any health care facility; OHCA due to drowning, exsanguination, or trauma; resuscitation efforts ceased due to a do-not-resuscitate order; resuscitation not attempted, such as in the presence of obvious signs of prolonged death; return of spontaneous circulation (ROSC) before EMS arrival; and incidents with missing time data required to identify IAT or COSR.

### Measures

The primary outcome of interest was the odds of IAT, defined as EMS departing the arrest scene prior to obtaining any ROSC (regardless of the duration of ROSC achieved). IAT differs from COSR, defined as on-scene ROSC or on-scene termination of resuscitation.

The primary exposure was neighborhood-level SVI percentile, which was stratified into quartiles. The SVI has been described elsewhere; a tabulated synopsis is provided in the eTable in Supplement 1.^34,35,36,37^ The SVI was first used to identify the most socioeconomically vulnerable areas during public health emergencies and natural disasters; however, it has also been used in public health research as an objective measure of a community’s relative social disadvantage.^38^ While we use the Centers for Disease Control and Prevention’s term *vulnerability*, we acknowledge that the word *marginalization* may more accurately reflect the structural inequities underlying these designations.^39,40,41^ The SVI is calculated using percentage estimates from the American Community Survey 5-Year Estimates for 2018-2022.^42^ It comprises 16 variables, each falling under 1 of 4 themes: socioeconomic status, household characteristics, racial and ethnic minority status, and housing type and transportation. For each neighborhood, percentile rankings for each of the 16 variables (against all other neighborhoods) are generated and added together. All neighborhoods are then ordered according to their summed percentiles to determine their overall SVI percentile ranking. Percentile rankings range from 0 to 100, with higher numbers indicating greater social vulnerability. We stratified overall SVI percentiles for each neighborhood into quartiles (<25th, 25th-50th, 51st-75th, and >75th percentiles). Thus, neighborhood SVIs below the 25th percentile represented the least vulnerable neighborhood quartile (SVI quartile 1), and neighborhood SVIs greater than the 75th percentile represented the most vulnerable neighborhoods (SVI quartile 4).

Following guidelines on reporting race and ethnicity in research, we kept the race and ethnicity data used to calculate the overall SVI to capture broader contextual factors impacting health outcomes.^43^ Inclusion of these data does not imply a causal association between race and ethnicity and neighborhood SVI, but instead it aims to identify inequities in health care and social determinants of health experienced by historically marginalized racial and ethnic groups. Neighborhood race and ethnicity estimates were generated from self-reported data in the American Community Survey.

Secondary outcomes included the association of SVI quartile with patient and encounter characteristics. Patient and encounter characteristics included patient age, sex, race and ethnicity (Hispanic or Latino, non-Hispanic Asian, non-Hispanic Black, Native American or Alaska Native, non-Hispanic Pacific Islander, non-Hispanic White, and other [not listed] or multiracial); bystander CPR; witnessed vs unwitnessed arrest; public (eg, public building, street, and place of recreation), private (eg, home and assisted living facility), or other scene location (eg, other specified place); shockable or nonshockable initial cardiac rhythm recorded by EMS; and urban vs rural scene location. These characteristics were recorded in the EHR directly by EMS clinicians.

### Statistical Analysis

We compared neighborhood, scene, cardiac arrest, and patient demographic characteristics between patients who received IAT vs COSR. To examine the association between neighborhood SVI and IAT, we used a marginal logistic regression model and generalized estimating equations to estimate model coefficients with an exchangeable working correlation structure, to account for potential clustering effects within EMS agencies. Because IAT was a common outcome in this cohort, adjusted odds ratios (ORs) were presented as measures of association and should not be interpreted as approximations of relative risk.

We selected covariates a priori based on prior literature.^6,10^ The model included neighborhood SVI quartiles and the EMS-entered patient and encounter characteristics. Patient race and ethnicity were included as measures and, in the model, as markers of differential experience of prehospital care. Due to small sample sizes, we combined adults identified by EMS as Native American or Alaska Native, non-Hispanic Pacific Islander, and other race or more than 1 race into 1 group and categorized it as other or multiracial. Encounters missing individual race or ethnicity data were excluded from race and ethnicity–stratified comparisons but were included in descriptive results of patient and encounter characteristics.

All statistical tests were 2-sided, with a threshold for statistical significance of *P* < .05. We provided 95% CIs, as appropriate. All statistical analyses were conducted between December 2023 and December 2024 using Stata version 18 (StataCorp LLC).

## Results

### Characteristics of Patients and Encounters for Out-of-Hospital Cardiac Arrest

**Figure 2.**
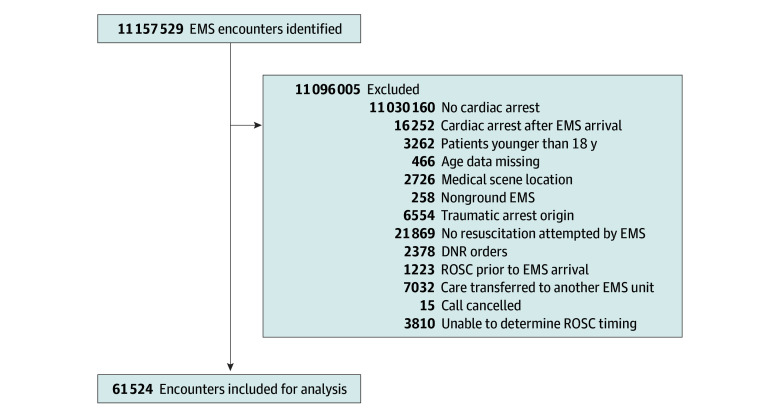
Flowchart of Inclusion of Encounters for Analysis DNR indicates do not resuscitate; EMS, emergency medical services; and ROSC, return of spontaneous circulation.

We analyzed 61 524 patient encounters from 2175 EMS agencies (Figure 2). Patients had a median (IQR) age of 65 (52-76) years and included 38 546 males (62.6%) and 22 864 females (37.1%). Race and ethnicity of these patients were as follows: 4134 Hispanic or Latino (6.7%), 1031 non-Hispanic Asian (1.6%), 11 662 non-Hispanic Black (18.9%), 163 Native American or Alaska Native (0.2%), 60 non-Hispanic Pacific Islander (0.1%), 38 348 non-Hispanic White (62.3%), and 138 other or multiracial (0.2%).

Of the OHCA events, 48 807 (79.3%) occurred in urban areas, 54 308 (88.3%) occurred in private locations, and 26 341 (42.8 %) were witnessed by bystanders. Bystander CPR was administered in 23 124 encounters (37.6%). A total of 10 830 OHCAs (17.6%) had an initial, shockable rhythm.

There were 17 173 encounters in the most socioeconomically vulnerable communities (SVI quartile 4), accounting for 28.0% of all cases, compared with 12 178 encounters (19.8%) in the least socioeconomically vulnerable communities (SVI quartile 1) (Table 1). There were 23 492 patients (38.2%) who received IAT and 38 032 patients (61.8%) who received COSR (Table 2). OHCAs in SVI quartile 4 had a higher occurrence of IAT compared with arrests in SVI quartile 1 (7052 [41.1%] vs 4000 [32.8%]; *P* < .001). Among the OHCAs occurring in public locations, 3602 patients (56.3%) received IAT. Of the OHCAs with an initial shockable rhythm, 5487 patients (50.7%) received IAT. The median (IQR) on-scene time before IAT was initiated was 20.7 (15.3-27.7) minutes. For arrests that achieved ROSC, the median (IQR) time between EMS arrival at the scene to ROSC was 27.8 (21.1-37.0) minutes for arrests that underwent IAT and 15.0 (9.6-20.8) minutes for arrests that underwent COSR.

**Table 1.** Patient and Encounter Characteristics by Neighborhood Social Vulnerability Index

Characteristic	Patients, No. (%)^a^				*P* value
	SVI quartile 1 (<25th percentile, least vulnerable neighborhoods)	SVI quartile 2 (25th-50th percentile)	SVI quartile 3 (51st-75th percentile)	SVI quartile 4 (75th percentile, most vulnerable neighborhoods)	
All	12 178 (19.8)	15 410 (25.1)	16 640 (27.1)	17 173 (28.0)	NA
Age, median (IQR), y	68 (56-78)	66 (54-77)	65 (52-75)	62 (50-74)	<.001
Sex					
Female	4220 (34.7)	5587 (36.3)	6353 (38.3)	6676 (38.9)	<.001
Male	7932 (65.3)	9791 (63.7)	10 252 (61.7)	10 476 (61.1)	
Race and ethnicity^b^					
Asian, non-Hispanic	310 (2.9)	270 (2.0)	254 (1.7)	196 (1.2)	<.001
Black, non-Hispanic	1162 (10.8)	1947 (14.1)	2900 (19.2)	5641 (35.7)	
Hispanic or Latino	498 (4.6)	813 (5.9)	1066 (7.1)	1733 (11.0)	
Native American or Alaska Native	23 (0.2)	35 (0.3)	43 (0.3)	62 (0.4)	
Pacific Islander, non-Hispanic	31 (0.3)	30 (0.2)	34 (0.2)	42 (0.3)	
White, non-Hispanic	8743 (81.1)	10 683 (77.5)	10 756 (71.4)	8095 (51.3)	
Other or multiracial^c^	12 (0.1)	11 (0.1)	21 (0.1)	16 (0.1)	
Urbanicity					
Urban	11 214 (92.1)	12 607 (81.8)	11 729 (70.5)	13 179 (76.7)	<.001
Rural	962 (7.9)	2799 (18.2)	4908 (29.5)	3994 (23.3)	
Scene location type					
Private	10 819 (88.9)	13 604 (88.3)	14 753 (88.7)	15 063 (87.7)	<.001
Public	1217 (10.0)	1593 (10.3)	1661 (10.0)	1892 (11.0)	
Other^d^	141 (1.2)	213 (1.4)	224 (1.4)	218 (1.3)	
Initial ECG					
Nonshockable	9583 (79.4)	12 322 (80.9)	13 580 (82.4)	14 514 (85.3)	<.001
Shockable	2491 (20.6)	2914 (19.1)	2895 (17.6)	2501 (14.7)	
Witnessed arrest					
No	6813 (56.0)	8629 (56.0)	9308 (55.9)	10 370 (60.4)	<.001
Yes	5365 (44.1)	6781 (44.0)	7332 (44.1)	6803 (39.6)	
Bystander CPR					
No	7434 (61.2)	9269 (60.3)	10 275 (61.9)	11 235 (65.6)	<.001
Yes	4723 (38.9)	6098 (39.7)	6330 (38.1)	5903 (34.4)	

Abbreviations: CPR, cardiopulmonary resuscitation; ECG, electrocardiogram; SVI, Social Vulnerability Index.

^a^ Numbers may not sum to 100% due to rounding error.

^b^ Race and ethnicity were determined by EMS clinicians and recorded in the electronic health record (EHR).

^c^ Other was recorded in the EHR as a category not otherwise listed and as more than 1 race. These groups were combined due to small numbers.

^d^ Other scene location was reported as other specified place in the EHR.

**Table 2.** Patient and Encounter Characteristics by Intra-Arrest Transport Status

Characteristic	Patients, No. (%)^a^		*P* value
	Received IAT	Received COSR	
All	23 492 (38.2)	38 032 (61.8)	NA
Neighborhood SVI quartile			
Quartile 1 (least vulnerable)	4000 (32.8)	8178 (67.2)	<.001
Quartile 2	5648 (36.7)	9762 (63.3)	
Quartile 3	6733 (40.5)	9907 (59.5)	
Quartile 4 (most vulnerable)	7052 (41.1)	10 121 (58.9)	
Age, median (IQR), y	64 (52-75)	66 (52-76)	<.001
Sex			
Female	8034 (35.1)	14 830 (64.9)	<.001
Male	15 425 (40.0)	23 121 (60.0)	
Race and ethnicity			
Asian, non-Hispanic	291 (28.2)	740 (71.8)	<.001
Black, non-Hispanic	4692 (40.2)	6970 (59.8)	
Hispanic or Latino	1616 (39.1)	2518 (60.9)	
Native American or Alaska Native	64 (39.3)	99 (60.7)	
Pacific Islander, non-Hispanic	20 (33.3)	40 (66.7)	
White, non-Hispanic	15 156 (39.5)	23 192 (60.5)	
Other or multiracial^b^	39 (28.3)	99 (71.7)	
Urbanicity			
Urban	17 362 (35.6)	31 445 (64.4)	<.001
Rural	6128 (48.2)	6580 (51.8)	
Scene location type			
Private	19 479 (35.9)	34 829 (64.1)	<.001
Public	3602 (56.3)	2802 (43.8)	
Other^c^	408 (50.4)	401 (49.6)	
Initial ECG			
Nonshockable	17 768 (35.5)	32 325 (64.5)	<.001
Shockable	5487 (50.7)	5343 (49.3)	
Witnessed arrest			
No	11 550 (32.8)	23 633 (67.2)	<.001
Yes	11 942 (45.3)	14 399 (54.7)	
Bystander CPR			
No	15 198 (39.7)	23 068 (60.3)	<.001
Yes	8234 (35.6)	14 890 (64.4)	
Response time, median (IQR), min^d^	6.3 (4.4-9.2)	6.1 (4.4-8.8)	<.001
On-scene time, median (IQR), min^e^	20.7 (15.3-27.7)	25.6 (19.7-32.7)	<.001

Abbreviations: COSR, continued, on-scene resuscitation; CPR, cardiopulmonary resuscitation; ECG, electrocardiogram; IAT, intra-arrest transport; SVI, Social Vulnerability Index.

^a^ Numbers may not sum to 100% due to rounding error. Categories without a missing subcategory did not have missing data.

^b^ Includes individuals whose race or ethnicity was recorded in the electronic health record (EHR) as a category not otherwise listed and as more than 1 race. These groups were combined due to small numbers.

^c^ Other scene location was reported as other specified place in the EHR.

^d^ Response time was defined as the time between emergency medical services (EMS) dispatch and EMS arrival at the arrest scene.

^e^ On-scene time was defined as the time between EMS arrival at the scene and patient transport from the scene.

### Factors Associated With Intra-Arrest Transport

After adjustment, the odds of receiving IAT significantly increased with higher neighborhood socioeconomic vulnerability (Table 3). Compared with cardiac arrest events occurring in neighborhoods in SVI quartile 1, events had greater odds of IAT in neighborhoods in SVI quartile 2 (adjusted OR [AOR], 1.17; 95% CI, 1.06-1.29), in SVI quartile 3 (AOR, 1.29; 95% CI, 1.14-1.44), and in SVI quartile 4 (AOR, 1.35; 95% CI, 1.15-1.57). The odds of IAT were greater for OHCAs among male patients (AOR, 1.13; 95% CI, 1.09-1.18), in rural locations (AOR, 1.59; 95% CI, 1.33-1.91), in public locations (AOR, 2.01; 95% CI, 1.83-2.21), in other locations (AOR, 1.62; 95% CI, 1.37-1.92), with shockable rhythms (AOR, 1.54; 95% CI, 1.44-1.65), and that were witnessed (AOR, 1.51; 95% CI, 1.42-1.60).

**Table 3.** Adjusted Odds of Intra-Arrest Transport

Variable	AOR (95% CI)
Neighborhood SVI quartile	
Quartile 1 (least vulnerable)	1 [Reference]
Quartile 2	1.17 (1.06-1.29)
Quartile 3	1.29 (1.14-1.44)
Quartile 4 (most vulnerable)	1.35 (1.15-1.57)
Age per 10-y increase	0.99 (0.97-1.00)
Sex	
Female	1 [Reference]
Male	1.13 (1.09-1.18)
Race and ethnicity	
Asian, non-Hispanic	0.69 (0.56-0.85)
Black, non-Hispanic	1.08 (0.97-1.22)
Hispanic or Latino	0.97 (0.76-1.23)
White, non-Hispanic	1 [Reference]
Other or multiracial^a^	0.74 (0.56-0.97)
Urbanicity	
Urban	1 [Reference]
Rural	1.59 (1.33-1.91)
Scene location type	
Private	1 [Reference]
Public	2.01 (1.83-2.21)
Other^b^	1.62 (1.37-1.92)
Initial ECG	
Nonshockable	1 [Reference]
Shockable	1.54 (1.44-1.65)
Witnessed arrest	
No	1 [Reference]
Yes	1.51 (1.42-1.60)
Bystander CPR	
No	1 [Reference]
Yes	0.82 (0.78-0.86)

Abbreviations: AOR, adjusted odds ratio; CPR, cardiopulmonary resuscitation; ECG, electrocardiogram; SVI, Social Vulnerability Index.

^a^ Includes individuals whose race or ethnicity was recorded in the electronic health record (EHR) as a category not otherwise listed (based on emergency medical services clinician impression) and as Native American or Alaska Native, non-Hispanic Pacific Islander, or as more than 1 race. These groups were combined due to small numbers.

^b^ Other scene location was reported as other specified place in the EHR.

## Discussion

In this large, geographically diverse analysis, patients with OHCA in the most socioeconomically vulnerable neighborhoods were more likely to receive IAT, a practice associated with poor survival outcomes.^16^ The higher prevalence of IAT, compared with COSR, that we observed among patients with OHCA in a public location and patients with an initial shockable rhythm, aligns with findings reported in previous studies.^17,24,44,45^ Additionally, we found that IAT was less prevalent than COSR among non-Hispanic Black patients. These findings are also consistent with prior studies reporting no increased IAT risk among this patient demographic.^46,47^

Several potential explanations exist for the differences in EMS transport practices by neighborhood SVI. First, qualitative studies found that EMS personnel report multiple factors affecting their decision to perform IAT, including numerous on-scene bystanders, communication challenges, and perceiving the scene as unsafe.^14,44^ These factors may be more prevalent in higher SVI neighborhoods. Scene crowding or the presence of multiple bystanders may lead EMS personnel to opt for IAT, which is supported by our finding that a public location doubled the odds of IAT. For example, neighborhoods with a higher prevalence of multioccupancy buildings and residences with group living quarters can lead to a higher SVI. Other studies have reported that communication difficulties between EMS personnel and bystanders affect EMS transport decisions, with 1 study showing shorter on-scene EMS times when there was language incongruency between EMS personnel and bystanders.^48,49,50^ Because language proficiency is a component of the SVI, neighborhoods with higher prevalences of individuals with limited English proficiency can have a higher SVI. Moreover, perceived scene safety may play a role in transport decisions.^44^ Various contextual factors, including neighborhood characteristics, personal experience, and law enforcement presence might alter assessments of scene safety.^44^ While essential to ensuring EMS and patient safety, these assessments may inadvertently introduce biases and affect transport decisions.

Second, EMS resource considerations, an EMS team’s overall level of training, and ambulance availability may be associated with IAT decision-making.^51^ Areas with EMS with volunteer staff may favor IAT to counterbalance their limited skill in performing advanced cardiac life support on scene.

Third, EMS protocols for transporting patients with OHCA without ROSC vary, and there is a lack of universally adopted guidelines.^52^ As individual states regulate EMS agencies, state-level and agency-level organizational factors and policies may contribute to the variation in IAT practices. For example, a review of 104 publicly accessible EMS protocols for adult OHCA transport expectations across 43 states found that 26% of protocols did not specify when transport should be initiated, 52% specified providing transport after ROSC, 7% stated patients should be transported after 20 minutes of COSR, and the remaining specified other directives.^52^ Thus, variable agency-level policies, if present, likely contribute to the observed differences in prehospital IAT practices for OHCA. In the absence of official directives, informal practice culture, which may be more susceptible to biases, may affect transport decisions. These complex decision-making processes underscore the intersectionality of health care system infrastructure, geographic disparities, and emergency response strategies.

Our finding that IAT occurs more often in neighborhoods with lower socioeconomic status suggests that structural and systemic inequities contribute to socioeconomic disparities in OHCA-related clinical outcomes. Neighborhoods characterized by lower socioeconomic status experience a confluence of challenges, including limited health care services and access. For instance, neighborhoods with lower socioeconomic status face chronic underfunding of health care resources, which may be associated with fewer EMS units, longer EMS response times, and reduced availability of advanced medical interventions. These resource limitations are considerations in EMS transport decisions if EMS teams must navigate high-demand, low-resource environments. Initiatives that expand EMS capabilities and enhance EMS personnel’s awareness of the clinical impact of IAT, particularly in socioeconomically marginalized areas, can be prioritized to address socioeconomic-based disparities in OHCA survival outcomes. Additionally, initiatives that address biases within the health care system and promote cultural humility may advance equitable prehospital care delivery.

### Limitations

Among its limitations, our study had a retrospective design and used data collected as part of routine clinical care. The study population included encounters with missing data; however, less than 10% of any single variable had missing data. Reasons for pursuing IAT or COSR were not documented in the EHR; therefore, our discussion of results was based on content knowledge and prior literature. However, the results describe an apparent disparity in prehospital transport practices by neighborhood socioeconomic vulnerability, which can inform hypothesis generation for future studies. Our definition of IAT as EMS transport occurring before any ROSC duration may also underestimate the true prevalence of IAT in this cohort given that other studies report that up to 30% of events achieving ROSC re-arrest on scene and may subsequently receive IAT.^53^ Therefore, the true prevalence of IAT in our study may be higher. Our IAT definition also depended on EMS departure from the scene, although in practice IAT begins during the minutes when a patient is being prepared for transport and placed in the ambulance. We were unable to account for this scene-evacuation period as such times were not captured in the ESO dataset.

Finally, our analysis did not include characteristics of the resuscitations themselves (eg, resuscitation duration), which could affect the benefit of IAT or COSR. Further investigations into resuscitation quality may further contextualize these results. Additionally, we did not include OHCA survival outcomes. This decision was intentional, as prior research has demonstrated a robust association between IAT for OHCA and decreased survival.^16^ Instead, we chose to investigate a plausible mechanism underlying the disparities in OHCA survival by assessing the implications of neighborhood-level characteristics for IAT practice, an important intermediate outcome along the OHCA chain of survival.

## Conclusions

In this national cohort study of EMS-treated OHCA, we found that patients in neighborhoods with greater socioeconomic vulnerability had progressively higher odds of receiving IAT, a practice associated with worse OHCA clinical outcomes. These findings highlight a potential structural contributor to disparities in OHCA outcomes and underscore the need for further investigation into how neighborhood context affects prehospital decision-making and patient outcomes. Future research should explore the mechanisms underlying these associations and evaluate interventions aimed at promoting equitable, evidence-based EMS practices.

## Notes

Supplement 1. **eTable.** The Centers for Disease Control and Prevention and Agency for Toxic Substances and Disease Registry Social Vulnerability Index

Supplement 2.
Data Sharing Statement
