# Supplementary material for: Disparities in Emergency Medical Services Intra-Arrest Transport by Neighborhood Socioeconomic Vulnerability
Source: JAMA Netw Open. 2026 Apr 3;9(4):e263764. doi: 10.1001/jamanetworkopen.2026.3764 (PMC13049492; doi:10.1001/jamanetworkopen.2026.3764)
Supplement: Supplement 1. — eTable. The Centers for Disease Control and Prevention and Agency for Toxic Substances and Disease Registry Social Vulnerability Index [file jamanetwopen-e263764-s001.pdf]

Supplemental Online Content

Hewlett MM, Crowe RP, Ager EE, Ford JS, Mercer MP, Hsia RY. Disparities in emergency medical services intra-arrest transport by neighborhood socioeconomic vulnerability. *JAMA Netw Open*. 2026;9(4):e263764. doi:10.1001/jamanetworkopen.2026.3764

eTable. The Centers for Disease Control and Prevention and Agency for Toxic Substances and Disease Registry Social Vulnerability Index

This supplemental material has been provided by the authors to give readers additional information about their work.

© 2026 Hewlett MM et al. *JAMA Network Open*

eTable: The Centers for Disease Control and Prevention and Agency for Toxic Substances and Disease Registry Social Vulnerability Index

Overall Vulnerability	
Socioeconomic Status	Individuals Below 150% of the Federal Poverty Level
	Civilians (Aged ≥ 16 years) Unemployed
	Housing Cost Burden: Occupied housing units with annual income of < $75,000 (≥ 30% of income spent on housing costs)
	Persons (Aged ≥ 25 years) with no High School Diploma
	Uninsured in the Total Civilian Noninstitutionalized Population
Household Characteristics	Persons Aged ≥ 65 Years
	Persons Aged ≤ 17 Years
	Civilian Noninstitutionalized Population with a Disability
	Single-Parent Households with Children Aged ≤ 18 years
	Persons (Aged ≥ 5 years) who Speak English "Less Than Well"
Racial and Ethnic Minority Status[a]	American Indian and Alaska Native, Not Hispanic or Latino; Asian, Not Hispanic or Latino; Black and African American, Not Hispanic or Latino; Hispanic or Latino (of any race); Native Hawaiian and Other Pacific Islander, Not Hispanic or Latino; Other Races, Not Hispanic or Latino; Two or More Races, Not Hispanic or Latino
Housing Type & Transportation	Housing in Structures with ≥ 10 Units
	Mobile Homes
	Occupied Housing Units with More People than Rooms
	Households with No Available Vehicle
	Persons in Group Quarters

Adapted from the Agency for Toxic Substances and Disease Registry.
[a]Other Races, Not Hispanic or Latino defined by the American Community Survey as comprising of individuals who self-identify as a race other than the categories provided.

© 2026 Hewlett MM et al. *JAMA Network Open*
