# Supplementary material for: Disparities in Emergency Medical Services Intra-Arrest Transport by Neighborhood Socioeconomic Vulnerability
Source: JAMA Netw Open. 2026 Apr 3;9(4):e263764. doi: 10.1001/jamanetworkopen.2026.3764 (PMC13049492; doi:10.1001/jamanetworkopen.2026.3764)
Supplement: Supplement 2. — Data Sharing Statement [file jamanetwopen-e263764-s002.pdf]

Data Sharing Statement

Hewlett. Disparities in Emergency Medical Services Intra-Arrest Transport by Neighborhood Socioeconomic Vulnerability. *JAMA Netw Open*. Published April 03, 2026. doi:10.1001/jamanetworkopen.2026.3764

Data
Data available: No

Additional Information
Explanation for why data not available: Data from the ESO Data Collaborative are available through a proposal process. More information can be found at www.eso.com/data-and-research.
